# An improved butanol-HCl assay for quantification of water-soluble, acetone:methanol-soluble, and insoluble proanthocyanidins (condensed tannins)

**DOI:** 10.1186/s13007-017-0213-3

**Published:** 2017-08-02

**Authors:** Philip-Edouard Shay, J. A. Trofymow, C. Peter Constabel

**Affiliations:** 1 Department of Biology & Centre for Forest Biology, University of Victoria, P.O. Box 3020, STN CSC, Victoria, BC V8W 3N5 Canada; 2 Pacific Forestry Centre, Canadian Forest Service, Natural Resources Canada, Victoria, BC Canada

**Keywords:** Tannin, Proanthocyanidins, Leaf litter, Nitrogen stress, Polyphenol, Flavonoid, Poplar, Douglas-fir

## Abstract

**Background:**

Condensed tannins (CT) are the most abundant secondary metabolite of land plants and can vary in abundance and structure according to tissue type, species, genotype, age, and environmental conditions. Recent improvements to the butanol-HCl assay have separately helped quantification of soluble and insoluble CTs, but have not yet been applied jointly. Our objectives were to combine previous assay improvements to allow for quantitative comparisons of different condensed tannin forms and to test protocols for analyses of condensed tannins in vegetative plant tissues. We also tested if the improved butanol-HCl assay can be used to quantify water-soluble forms of condensed tannins.

**Results:**

Including ~50% acetone in both extraction solvents and final assay reagents greatly improved the extraction and quantification of soluble, insoluble and total condensed tannins. The acetone-based method also extended the linear portion of standard integration curves allowing for more accurate quantification of samples with a broader range of condensed tannin concentrations. Estimates of tannin concentrations determined using the protocol without acetone were lower, but correlated with values from acetone-based methods. With the improved assay, quantification of condensed tannins in water-soluble forms was highly replicable. The relative abundance of condensed tannins in soluble and insoluble forms differed substantially between tissue types.

**Conclusions:**

The quantification of condensed tannins using the butanol-HCl assay was improved by adding acetone to both extraction and reagent solutions. These improvements will facilitate the quantification of total condensed tannin in tissues containing a range of concentrations, as well as to determine the amount in water-soluble, acetone:MeOH-soluble and insoluble forms. Accurate determination of these three condensed tannin forms is essential for careful investigations of their potentially different physiological and ecological functions.

## Background

Condensed tannins (CT), also known as proanthocyanidins, are the most abundant secondary metabolite of land plants. They can be found in many species but are most prevalent in woody plants, where they accumulate in most major tissues including leaves, bark, and roots [1]. Condensed tannins are polymers of flavan-3-ols, and end products of the well-characterized flavonoid pathway [2]. High concentrations of CTs have been shown to provide protection against vertebrate herbivores due to binding of dietary protein in the gut [2]. A defensive function against lepidopteran insect herbivores has also been proposed, but is more likely based on the pro-oxidant nature of tannins [2]. Condensed tannins demonstrate broad anti-microbial properties in vitro [3], and CT concentrations have been correlated with reduced rates of pathogen infection in the field [4]. Other hypothesized functions of CTs include roles in roots as protection against metal toxicity, and in soil as mediators of decomposition and nutrient cycling by microbes [5].

Condensed tannin structures can show subtle variation in the degree of polymerization, hydroxylation of the flavonoid B-ring, and stereochemistry [6]. Both abundance and structure of CTs can vary depending on tissue type, species, genotype, age and environmental conditions [7–14]. Much of this variation still awaits functional characterization [2], but is likely to be associated with differences in biological activity [6, 15, 16]. Furthermore, CTs extracted from a given species and tissue will contain a mixture of CTs with subtle structural differences, for example a broad range of polymer lengths. The individual compounds are difficult to separate using chromatographic methods, which has made precise determination of individual structures difficult. Nevertheless, methods that rely on depolymerization in the presence of thiol or phloroglucinol give useful structural information and average subunit composition [17, 18]. In addition, sophisticated LC–MS/MS methods which rely on the in-source fragmentation of oligomeric and polymeric CTs have been developed recently. These also provide data on subunit composition and degree of polymerization in complex extracts [19].

The most common and straightforward method for quantifying CTs has been the butanol-HCl method developed by Swain and Hillis [20] and improved by Porter et al. [21]. This involves depolymerization of the polymer in acid and conversion of the monomers to anthocyanidin, which can be spectrophotometrically quantified. Structural differences in CTs from different sources can lead to differences in reactivity to this assay; therefore, a purified standard of CT isolated from the same species and tissues to be analyzed is essential if absolute quantitation is required. However, using the standard for quantifying CT across species and tissues, as done in this study, may be more workable and still allows for relative quantitation and comparisons between treatments. An advantage of the butanol-HCl method is that it permits a direct quantification of all CT fractions, i.e., water-soluble, organic solvent-extractable, and unextractable (functionally insoluble) CTs [17, 22]. Depending on the solvent used, these insoluble CTs typically make up between 10% and 50% of total CT content, and in some tissues can constitute 90% of total CTs [23]. Nevertheless, they are poorly investigated and often ignored [17, 22, 24]. What renders these fractions insoluble is not well-understood, but greater polymer length has been associated with a greater proportion of insoluble CT [17].

In leaf litter, cross-linking of CTs during cell death and senescence has been suggested [7, 25]. The proportion of insoluble CTs in foliar litter fall is thus dynamic, and likely also differs between species, but is rarely reported [26–28]. For example, the impact of CTs on decomposition and nitrogen mineralization in soil has been extensively investigated, but differential roles for soluble and insoluble components of the CTs are not easily defined [10, 15, 25, 27, 29–32]. Preliminary data suggest that these tannin fractions have different stabilities in soils [33], and a method that can efficiently assay and compare these is needed for ecological studies. The butanol-HCl assay can be used directly on plant material or on solvent-extracted residue, providing a measure of both soluble and insoluble CT fractions from the same sample. Associating potential biological functions to soluble and insoluble components of CT is difficult since these are defined by the solvents used for extraction. Furthermore, water soluble tannins may be ecologically more relevant than solvent soluble fraction, yet CTs are rarely quantified in water extracts using the butanol-HCl method [14]. One reason is likely the assay’s sensitivity to the presence of water [21]. Typically, the analysis of CT using the butanol-HCl protocol is carried out on fractions in aqueous acetone [7, 26, 32, 34] or methanol [9, 35]. Where quantification of CT in insoluble forms is performed, available approaches have not applied the appropriate solvent concentrations needed for a direct comparison of soluble and insoluble fractions of CTs [36] and for exhaustive extraction [35, 37]. Consolidating disparate extraction and assay conditions was a major motivation for developing our modified method.

Recent improvements of the butanol-HCl protocol for CT quantification have separately enhanced the extraction of soluble CT forms by optimizing solvent concentrations and heating temperatures [38] and improving quantitation of total CT [i.e. soluble and insoluble forms; 36] by modifying reagent concentrations. However, these modifications have not yet been combined into one efficient method. The objectives of our study, therefore, were to combine improvements of the butanol-HCl assay described by Mané et al. [38] and Grabber et al. [36] into one efficient protocol. Our aim is to improve solvent extraction of CT, and facilitate direct quantitative comparison of soluble and insoluble fractions of CTs forms from different types of tissues and samples, in particular foliar litter. Additionally, we show how our methodological improvements allow for easy quantification of water-soluble CTs.

## Methods

### Condensed tannin standards

CT standards were purified from leaves of *Populus tremula* × *tremuloides* [INRA clone 353-38; 34] by the method of Fierer et al. [40] using chromatography on Sephadex LH-20 resin with the following changes: sample pre-treatment with hexane was omitted, and the dried crude extract was resuspended in 50% EtOH, and filtered on 0.45 μm polyvinylidene difluoride membrane (EMD Millipore, Germany) rather than treating with ethyl acetate. Elution of purified CT from the Sephadex LH-20 column using 70% aqueous acetone was only carried out after successive washing of the column with 80% EtOH yielded fractions with a UV absorbance at 280 nm of less than 0.5 absorbance units (AU). The CT standard was characterized and checked for purity by NMR as described by Preston and Trofymow [14].

### Plant material

Assay development and improvements were performed using naturally abscised poplar leaves (*Populus angustifolia*) and Douglas-fir (*Pseudotsuga menziesii*) needles. Naturally abscised Douglas-fir needle litter fall (hereafter litter) was collected from the Shawnigan Research Forest, Vancouver Island, British Columbia, Canada [39]. Naturally abscised poplar leaf litter was from a common garden at the Ogden Nature Center (Ogden, UT, USA) kindly provided by Dr. Thomas Whitham and the Cottonwood Research Group at Northern Arizona University. Poplar-leaf litter was pooled from trees with known leaf chemistries in order to set leaf litter treatments with low and high CT concentrations. To generate poplar or Douglas-fir foliar litter with high and low nitrogen concentrations, samples were sprayed with either a glutamine solution or distilled water.

Fresh leaf and root tissues for testing the assay improvements on samples with known CT content were obtained from the University of Victoria’s Glover Greenhouse and the Constabel laboratory. Fresh leaf tissues consisted of greenhouse grown *Populus tremula* × *tremuloides* (INRA clone 353-38) and MYB134-overexpressing high CT line of the same hybrid [41]. Fresh roots and leaf tissues from untransformed (WT line 353) plants grown under N-limited conditions to induce CT synthesis were also tested. All fresh material was first flash-frozen in liquid N, ground using mortar and pestle, and lyophilized prior to analysis. For the experiment comparing tissue homogenization, a hammer mill model (Polymix PX-MFC 90D, Kinematica, Switzerland) was also used.

### Extraction and assay conditions

**Fig. 1:**
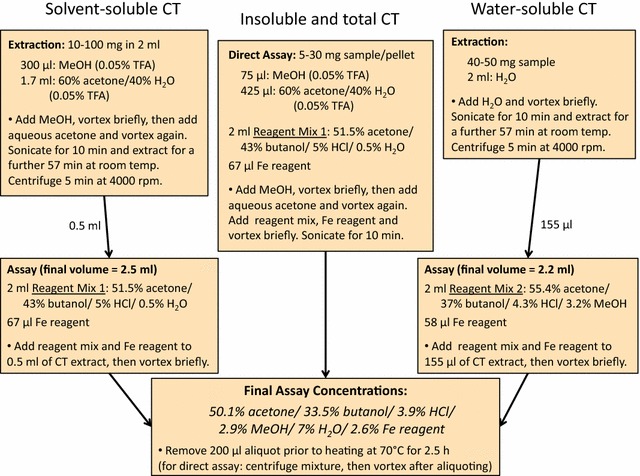
Flow diagram of solvent and reagent concentrations during assay preparation. Solutions are listed in the order in which they are added for assaying total-soluble, water-soluble or insoluble (or total) condensed tannins. Respective volumes used are also listed to clarify changes in concentrations. All listed ratios represent v/v and concentrated HCl (~12 N). The iron reagent was 2% w/v NH_4_Fe(SO_4_)_2_ and H_2_O in 2 N HCl

**Fig. 2:**
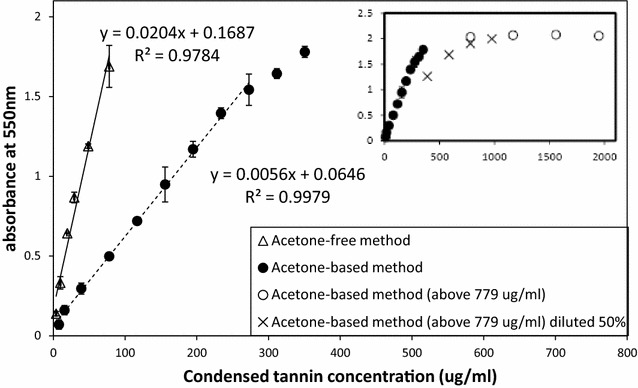
Standard curves generated with purified poplar condensed tannin. *Each point* in the main panel represents the mean of at least two independent values (±SE). Best fit lines were drawn through all data when using acetone-free method (74% butanol/3.9% 12 N concentrated HCl/15.6% MeOH/3.9% H_2_O/2.6% Fe-reagent) and between absorbance values of 0.07–1.54 for acetone-based method (50.1% acetone/33.5% butanol/3.9% 12 N concentrated HCl/2.9% MeOH/7% H_2_O/2.6% Fe-reagent). The preferred absorbance range for CT quantification when using acetone-based method was from 0.158 to 1.247 (abs. value ± 3 × SE). In the inset, samples with the four most concentrated CT standards (*open circles*) were diluted by 50% (*crosses*) and absorbance readings repeated. The comparison to equivalent concentrations of undiluted extract (*solid circles*) indicates that a discrepancy in predicted and actual absorbance values, suggesting that post-assays dilution will lead to erroneous results

The assay conditions and method are summarized in Fig. 1. Butanol-containing reagents were prepared on the same day they were used. The ratio of tissue weight to solvent (or assay reagent) depended on the tissue type and is described under Results, and sample amounts were adjusted to keep absorbance readings within the preferred range (Fig. 2). In some cases where CT concentrations in tissues were extremely high, solvent and reagent volumes were increased in order to avoid using too little sample.

To obtain solvent-extractable CTs, an appropriate volume (300 μl) of MeOH acidified with 0.05% trifluoroacetic acid (TFA) was added to dried tissue samples first. The slurry was briefly vortexed (<5 s) prior to adding 1.7 ml 60% aqueous acetone acidified with 0.05% TFA. Thus, the final solvent mixture consisted of 51% acetone, 34% MeOH, and 15% dH_2_O, the whole acidified with 0.05% TFA. The slurry was briefly vortexed, sonicated for 10 min and extracted for a further 57 min at room temperature (67 min total). During the extraction period, sample tubes were mixed by vortexing periodically (3 times for 5 s). Extracts were then clarified by centrifugation (5 min at 4000 rpm) and supernatants (CT extracts) removed and placed into fresh tubes.

To assay soluble CTs, 0.5 ml of extract was mixed with 2 ml of butanol-containing reagent (51.5% acetone/43% butanol/5% 12 N concentrated HCl/0.5% H_2_O) and 67 μl of Fe-reagent (2% w/v FeNH_4_(SO_4_)_2_ in 2 N HCl). The final assay mixture, containing both sample and assay solutions in a 2.5 ml volume, was thus comprised (v/v) of 50.1% acetone, 33.5% butanol, 3.9% 12 N concentrated HCl, 7% dH_2_O, 2.9% MeOH, and 2.6% Fe-reagent in a total volume of 2.5 ml (For simplicity, we do not include the additional H_2_O found in concentrated HCl and in the Fe-reagent in this breakdown; with this, the actual total H_2_O content approaches 12%). Aliquots (200 μl) of the final assay mixture of were removed to be read as non-heated controls. Assay samples were heated to 70 °C for 2.5 h, allowed to cool to RT, and the absorbance read at 550 nm using a Victor™ X5 Multi-label plate reader (PerkinElmer Inc.). To determine CT concentration, absorbances from unheated aliquots were subtracted from heated samples. When assaying water extracts for CTs, the proportion of assay reagent components, as well as volumes of extract and iron reagent, were adjusted to give the same water and solvent concentrations in the final assay mixture as for solvent extracted CTs (see Fig. 1). Maintaining a consistent proportion of water in the final assay mixture throughout is critical, as water is known to dramatically influence anthocyanidin formation in this assay [18, 21].

Insoluble CTs were determined directly on the centrifuged tissue pellets after extraction of soluble CTs. First, 75 μl MeOH (with 0.05% TFA) was added to pellets and vortexed, prior to adding 425 μl of 60% aqueous acetone (with 0.05% TFA). The remaining components of the assay reagent mix were then added, and the assay carried out directly on the suspended pellets (Fig. 1). The direct assay for total CTs was carried out in the identical manner. For assays performed directly on pellets or samples, mixtures were centrifuged 5 min at 4000 rpm prior to taking non-heated and heated aliquots for absorbance readings, and pellets re-suspended by vortex mixing before heating.

For comparison, samples were also assayed for CT concentration using 80% MeOH for soluble-CT extraction and the standard butanol-HCl assay reagent with methanol (74% butanol/3.9% 12 N concentrated HCl/15.6% MeOH/3.9% H_2_O/2.6% Fe-reagent (v/v). CT analyses are often carried out on MeOH extracts, which are more compatible with other analytical methods such as HPLC. To our knowledge, only one study of butanol-HCl assays comparing solely MeOH extracts with assays of acetone extracts have been made [42].

Statistical analyses were performed using R-statistics version 3.1.2 [43]. Purified CT standard curves were analysed using linear regression and r^2^. ANOVA and Kruskal–Wallis H-test were used to compare CT quantification in samples ground in liquid N_2_ using mortar and pestle or mechanically milled. Pearson correlations and linear regressions were used to compare one- versus two-step assay approaches as well as acetone-based versus acetone-free methods for quantification of total CT. Pearson correlations and ANOVA were used to assess differences in CT content between tissue types.

## Results

In order to more effectively measure CTs in a diversity of plant samples, but foliar litter in particular, we incorporated several modifications into one method. We used the solvent ratios of 51:34:15 (acetone:water:methanol) previously optimized by Mané et al. [38] in order to maximize extraction of the soluble CTs. The inclusion of 50% acetone in the final assay reagent, as per Grabber et al. [36], also improved the assay by extending the linear range of the assay response of purified poplar CT (Fig. 2). It also reduced the slope of the standard curve. This is in contrasts with results shown by Grabber et al. [36], but could be due to trends associated with the different H_2_O concentrations in our assays with and without acetone [21]. We also checked whether diluting a standard sample post-assay, i.e. after the heat treatment, would provide the same result as a dilution prior to the assay, but note that this leads to underestimates (Fig. 2 inset).

**Fig. 3:**
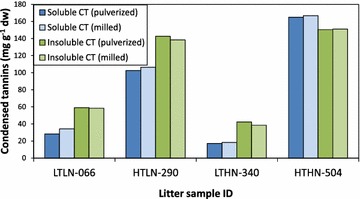
Quantification of soluble and insoluble condensed tannin using hammer-milled or liquid N_2_-pulverized samples. *Each column* represents an individual assay on a unique litter sample, from a larger-scale litter decay study; *p* > 0.05, ANOVA or Kruskal–Wallis H-test depending on data normality

When we tested the length of the incubation time at 70 °C, we found that absorption values and the slope of integration curves continued to increase for at least 2.5 h. However, after 2.5 h of heating, the average absorbance increased by 2.7% in the last 30 min of heating, and less than one-half of the increase measured in the previous 30 min (data not shown). Therefore, 2.5 h was used as the standard heating time. We observed minimal to no color development in non-heated controls at room temperature over 2.5 h (data not shown). Nonetheless, we read the absorbance of unheated controls immediately (<5 min) after pipetting aliquots in order to reduce the potential for anthocyanidin production and evaporation losses. Homogenizing samples in liquid-N_2_ by mortar and pestle did not improve the quantification of soluble and insoluble CT compared to hammer mill homogenization, suggesting access to solvent did not limit extraction (Fig. 3).

**Fig. 4:**
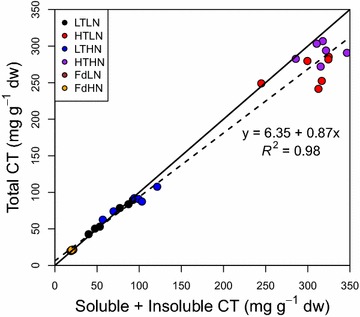
Comparison of total condensed tannin quantification using one- or two-step assay approaches. Condensed tannin values obtained using the two-step assay (soluble + insoluble CT) and the one-step method (direct assay for total CTs) are compared using Pearson’s correlation value and the best fit linear response (*dashed line*). *Colors* represent different leaf-litter chemistries, namely Douglas-fir (Fd) and poplar litter with low and high nitrogen concentrations (LN and HN) and CT concentrations (LT and HT, poplar only). The *black line* represents a 1:1 response

One of our goals was to adapt and test the method on the solvent-insoluble CT fraction, since insoluble CTs appear to be of particular ecological relevance in foliar litter. Total CT concentrations, comprising both soluble and insoluble fractions, were determined by using the direct assay on the ground tissue (Fig. 4). The resulting concentrations were compared to those obtained with the improved method and assaying acetone-extracted soluble tannins first, and then assaying the insoluble CTs in the remaining pellet (two-step assay). Tannin concentrations determined by the direct assay method were highly correlated with concentrations determined using the two-step assay approach (Pearson correlation *r* = 0.99). However, the direct assay method generally led to slightly smaller estimates of CT concentration (Fig. 4).

**Fig. 5:**
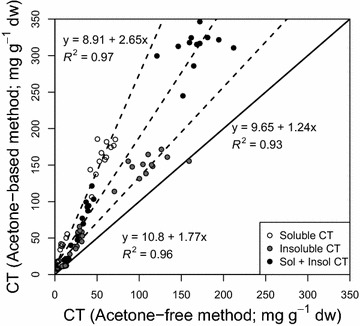
Comparison of two-step condensed tannin quantification using butanol-HCl assays with or without acetone. *Points* represent concentrations of soluble or insoluble CTs, or the sum of both two forms (Sol + Insol CT). Sample to solvent concentrations were ~1 mg ml^−1^ for the protocol without acetone, and 5–20 mg ml^−1^ for the acetone containing protocol. *Top, middle and bottom* equations correspond to soluble, insoluble, and soluble + insoluble CT quantifications

We compared results obtained using the improved acetone-containing method with those measured using 80% MeOH as a solvent and a butanol-containing reagent without acetone. Despite being highly correlated (*r* = 0.96 and 0.98 for soluble and insoluble fractions respectively), soluble and insoluble CT concentrations measured using the acetone-based assay were on average 3× and 1.4× greater, respectively, than concentrations measured using the former assay (Fig. 5). In other words, including acetone in both extraction solvent and final assay reagent appeared to lead to a more exhaustive extraction of CTs in foliar litter, while also improving the accuracy of CT standard curves across greater concentration ranges.

**Fig. 6:**
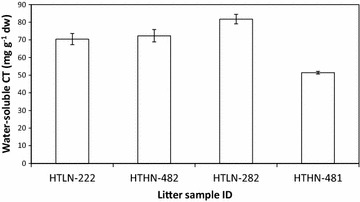
Replication of water-soluble condensed tannin quantification. *Each bar* represents an average of three independent assays (i.e. performed on different days; ±SE) performed on four litter samples (designated by a unique number) representing high-CT (HT) poplar litters with high (HN) and low N (LN)

The quantification of water-soluble CT fractions was highly replicable (Fig. 6). However, the high sample to solute ratio required for the assay (25–200 mg ml^−1^) prevented the quantification of residual CT in pellets after H_2_O extraction, due to the high volume of butanol-containing reagent needed to maintain absorbance values within spectrophotometric detection limits.

**Fig. 7:**
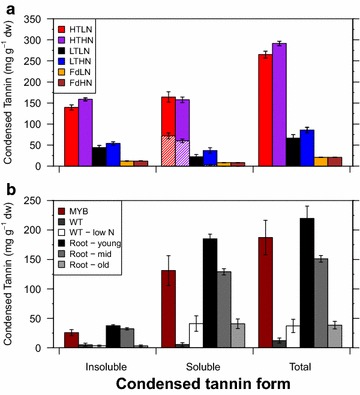
Average condensed tannin concentrations in plant tissues. **a** Tissues include poplar (n = 6) and Douglas-fir (n = 4) litter with high or low nitrogen (LN and HN) and condensed tannins (LT and HT, poplar only). **b** Fresh poplar tissues from greenhouse-grown poplar. Leaf tissue is from wild-type *P. tremula* × *tremuloides* (WT, n = 4), a high-CT (MYB115 over-expressing) transgenic *P. tremula* × *tremuloides* (MYB, n = 4), and a nitrogen-deprived plant (low N; n = 3). White roots within or farther than 5 mm of the root tip were classified as young or mid, respectively, while visibly brown roots were classified as old. For litter samples, condensed tannins in water-soluble forms are designated by hatching

We next tested the improved assay methods for total soluble (water- and acetone:MeOH-soluble forms) and insoluble CTs concentration on a set of samples consisting of high- and low-tannin litter (poplar and Douglas-fir) as well as poplar tissue samples. The latter included leaves from high-CT transgenic poplars [44] as well as leaves and roots from N-deficient plants, as these also show elevated tannins. Soluble and insoluble CTs were correlated across both species when considering only litter samples (Fig. 7a; *r* = 0.95; *p* < 0.001), and across tissue types when considering only fresh poplar tissue (Fig. 7b; *r* = 0.95; *p* < 0.001). By contrast, the ratios of soluble CT to total CT differed significantly between naturally abscised and fresh tissues (0.43 and 0.80 on average, respectively; *p* < 0.001; Fig. 7). In fresh plant tissues, CT was mostly in soluble form, but the amount and proportion of soluble to total CT also varied with N status (Fig. 7b). By contrast, foliar litter from both species had a significant insoluble CT component, comprising almost 50% of total CTs (Fig. 7a). Water-soluble CT concentration in foliar litter correlated better with acetone:MeOH-soluble CTs than the insoluble CT concentration (*r* = 0.94 and 0.90, respectively). Water-soluble CTs concentrations in fresh tissue was not measured.

The improved method was validated with several different available poplar tissue types and different stages of decaying foliar litter, to determine appropriate ratios of sample to solvent and sample to reagent for assays of soluble, insoluble, and total CTs (Table 1). The complete assay was repeated on each sample, incrementally adjusting sample to solvent (or reagent) ratios prior to heating, until the AU of heated solutions and the difference between heated and unheated solutions fell within the linear portion of our standard curve (without the need for dilution). Appropriate amounts of poplar tissue ranged from 5 to 10 mg ml^−1^ solvent for soluble CTs, and 20 mg ml^−1^ solvent for Douglas-fir litter. For the insoluble or direct methods, only small amounts of sample/pellet are needed, in particular for high-tannin tissues such as roots, where 0.6 mg ml^−1^ was found to be appropriate. Therefore, before applying these methods on different plant tissues, preliminary analyses should be done to determine appropriate sample to solvent ratios.

**Table 1:** Optimal sample concentrations (mg ml^−1^) for quantification of condensed tannins using improved acetone-based butanol-HCl assay

Samples	Species	Growing conditions	Tissue type	Soluble CT (sample/solvent)	Insoluble CT (pellet/reagent)	Total CT (sample/reagent)
Fresh leaf_MYB115/353(4)	*Populus tremula* × *tremuloides*	Greenhouse grown, well fertilized	Green leaf	5.0	1.3	0.6
Fresh leaf_WT353	*Populus tremula* × *tremuloides*	Greenhouse grown, well fertilized	Green leaf	10.0	7.8	3.9–9.7
Fresh leaf_Low N_WT 353	*Populus tremula* × *tremuloides*	Greenhouse grown, nitrogen limited	Green leaf	10.0	7.8	3.9–9.7
Roots (young)_Low N	*Populus tremula* × *tremuloides*	Greenhouse grown, nitrogen limited	Live root	5.0	1.3	0.6
Roots (mid-sized)_Low N	*Populus tremula* × *tremuloides*	Greenhouse grown, nitrogen limited	Live root	5.0	1.3	0.6
Roots (older)_Low N	*Populus tremula* × *tremuloides*	Greenhouse grown, nitrogen limited	Live root	10.0	2.6	1.3
Litter_LTLN	*Populus angustifolia*	Field grown	Abscised leaf	5–10	1.3–2.6	1.3
Litter_HTLN	*Populus angustifolia*	Field grown	Abscised leaf	5.0	1.3	0.6
Litter_LTHN	*Populus angustifolia*	Field grown	Abscised leaf	5–10	1.3–2.6	1.3
Litter_HTHN	*Populus angustifolia*	Field grown	Abscised leaf	5.0	1.3	0.6
Litter_FdLN	*Pseudotsuga menziesii*	Field grown	Abscised leaf	20.0	5.2	3.9
Litter_FdHN	*Pseudotsuga menziesii*	Field grown	Abscised leaf	20.0	5.2	3.9
Decayed litter_Poplar	*Populus angustifolia*	Field grown and decayed	Decayed abscised leaf	Not detected	19.5–39	11.7
Decayed litter_Douglas-fir	*Pseudotsuga menziesii*	Field grown and decayed	Decayed abscised leaf	39 to not detected	31–39	11.7

## Discussion

### Quantification of condensed-tannin forms using improvements on the butanol-HCl assay

The butanol-HCl assay for condensed tannins was improved using solvent concentrations and heating temperatures from Mané et al. [38] for better extraction of soluble tannins and applying reagent concentrations from Grabber et al. [36] that allow for quantitative comparison between CT forms. Our results suggest that solvent mixtures and protocols derived by Mané et al. [38] and Grabber et al. [36] were transferable to poplar and Douglas-fir tissues, therefore indicating broader application potential. Incorporating trifluoroacetic acid (TFA), MeOH, water, and ~50% acetone into extraction solvents for soluble CT assays, as well as including ~50% acetone in final assay reagents for assaying total CT, allowed for a more thorough CT quantification in foliar litter. As noted earlier [36], the 50% acetone in the final assay solution also eliminated quantification issues associated with the commonly observed ‘biphasic’ standard curve seen with the classic butanol-HCl protocol [21]. This is an additional advantage of the improved method.

We did not attempt re-optimize the earlier protocols and solvent ratios for our plant species and tissues, based on the assumption that poplar CTs are sufficiently similar in structure to those analyzed in the previous studies. Mané et al. [38] optimized their solvent mixture for grape seeds pulp, and skin extracts, with CTs having a mean degree of polymerization (DPM) of 2.9–39, prodelphinidin content of 0–14.5%, and galloylation at 1.1–9.5% depending on the tissues. Grabber et al. [36] developed their reagent mixture for use on two *Lotus* species differing mainly in procyanidin to prodelphinidin ratios (60:40 and 21:79), with no galloyl groups and polymers with average DPM from 5 to 38, depending on species. Poplar CTs vary in DPM from 2 to 28, with up to 50% prodelphinidins [12], and our purified poplar CT standard contained approximately 10% prodelphinidin and with an average DPM of 5.6 as verified by NMR [14, C. Preston, and C. P. Constabel, unpublished data]. Since both previous reports suggest that acidified mixtures with ~50% acetone are effective at removing fibre-bound CTs [36, 38], we are confident that this concentration is effective across a range of CT and tissue types. The consistently higher concentrations of soluble CT measured with ~50% acetone in both extraction solvent and reagent solution (Fig. 7) is likely due to the greater extractability of bound CT due to acetone/TFA, or the enhanced depolymerisation of CT complexes into flavan-3-ols. The increase in soluble CTs did not occur at the expense of insoluble CTs, which also increase with the new method. Therefore, the method appears to facilitate more efficient depolymerisation of CT bound to the litter matrix as well as in solution. Heating the assay tubes for 2.5 h was adequate for cleaving almost all of the CT polymers found in our samples, as additional incubation times had minimal effect. By contrast, quantification of CT using MeOH as an extraction solvent leads to underestimates but does provide reliable relative quantification of soluble CT concentration. It could thus be useful where relative quantification is the priority, and where methanolic extracts are preferred for additional downstream analyses such as HPLC or antioxidant tests.

The insoluble CT fraction may have been overestimated when performing the butanol-HCl assay directly on the residual pellet after extracting soluble CT (Fig. 4), due to remnant solvent in the pellet. Subtracting soluble from total CT values, with each assay done on separate subsamples, should lead to better quantification of the insoluble CT fraction. Washing pellets with MeOH prior to assaying insoluble CT [27] could also resolve this carry-over. In addition, we tested litter to solvent ratios for assaying both soluble and insoluble CT fractions to ensure that coloured anthocyanidin solutions were directly within the linear range of our standard curve. We note that dilution of reaction media after heating is sometimes carried out if absorbance values are too high [e.g. 35], but this should be avoided as it can lead to underestimates of CT concentrations (Fig. 2, inset).

The more effective extraction and depolymerisation of CT tightly bound to proteins or cell wall polysaccharides is most likely responsible for the greater proportion of insoluble CTs (~50%) we measured in our foliar litter samples compared to previous studies on foliar litter [26–28]. This suggests that the input of insoluble CTs into soil systems is greater than previously thought. By contrast, in fresh leaves, the proportion of insoluble CTs was only 10–20%. While these leaf samples are from a distinct poplar species grown under different environmental conditions, the much higher insoluble CT proportion in our litter could suggest that during senescence, soluble CTs become cross-linked and insoluble. Lindroth et al. [7] had previously suggested CT in poplar species undergo a shift from soluble to insoluble forms during senescence, especially in N-limited trees. By contrast, studies of developmental trajectories of CT in mangrove species do not show such increase in insoluble CT prior to senescence [28, 45]. A change in CT form during senescence could have important implications for litter decay, nutrient-cycling and other below-ground processes. Our assay improvements, together with methods for distinguishing protein and fibre-bound CT [46], will help in understanding the dynamics of soluble and insoluble CTs during development and senescence.

Slight modifications of the reagent concentrations used for the butanol-HCl assay allowed us to quantify the amount of water-soluble CT in leaf litter. This variation of our procedure releases an ecologically relevant form of CT, since we made extracts using room-temperature rather than hot water [14]. Water can solubilise low molecular weight phenols such as flavan-3-ol monomers, yet these are not converted to anthocyanidins during the butanol-HCl assay since they lack the carbocations resulting from interflanavoid bond cleavage [18, 21].

### Sources of error for the butanol-HCl assay

It is well established that the choice of a standard used for the butanol-HCl assay is critical, since structural differences of CTs from different species will influence reactivity and color formation. For example, differences in degrees of polymerization [6, 14, 17, 18, 37, 46] alters the ratio of extender to terminal subunits, which are not detected by the assay [21]. Our estimates of CT concentrations in *P. menziesii* needle litter could therefore be low, due to differences in degree of polymerization of CT from *P. menziesii* compared to *P. tremuloides* leaves [6, 13, 27], as we did not have access to a *P. menziesii* CT standard for this work. Choice of standards could also explain the slightly lower (2.1 vs. 4.3% dw) CT concentrations measured in our study compared to results by Preston et al. [27]. These authors used the same *P. menziesii* litter collection as we did, but quantified CT using purified CTs from *Abies balsamea*, a genus with shorter CT polymers than is typical for *P. tremuloides* [6, 13, 47]. Total CT concentrations in the *P. menziesii* litter used by Preston et al. [27] were indeed reduced to values similar to those obtained in our study when we recalculated concentrations using the poplar CT standard [14]. The absence of hydrolysable tannins in our poplar CT standard also avoids another common factor leading to overestimation of CTs [6, 35]. The high purity of our poplar CT standard [14] further avoided potential sources of error [36].

Estimates of CT concentrations in our poplar litter should be more accurate since we used a poplar CT standard; however, high variation in the degree of CT polymerization has been described in *Populus* [7, 12], and may have led to slight over-estimation of our comparatively more polymerized *P. angustifolia* species. We also note that discrepancies can arise when the CT not extracted from plant material using the same solvents as for the sample analysis. The degree of CT polymerization can affect their solubility and binding affinity [6, 18, 48], and thus their extractability. As a result, during the purification process, some smaller oligomeric CTs may have been eliminated [40], biasing the standard towards larger polymers. This could be prevented via the development of CT purification protocols encompassing water-soluble, MeOH-soluble, acetone-soluble and insoluble CT fractions for better representation of the range of CT forms present. However this issue may be difficult to resolve for the insoluble CTs, since to our knowledge, pure insoluble CT cannot be isolated [22] and is thus not included in typical purified CT standards [for details about extracts with insoluble CT, see ref. 24].

## Conclusions

Solvents have varying absorbance qualities and influence absorbance response curves to CTs. It is therefore important to maintain the same final reagent concentrations for all assays, whether analysing soluble, insoluble, or purified CTs so they can be directly compared. Our improvements on the butanol-HCl protocol allowed for a highly-replicable and more thorough quantification of both soluble and insoluble CT fractions in foliar litter and plant tissues. Our results show that the concentration of insoluble CTs in senesced foliar litter is greater than previously thought, and that water-soluble CT forms can make up a substantial proportion of foliar litter CT. Changes in the distribution of the CT forms during senescence may have important implications for above- and below-ground interactions.

## Abbreviations

CT: condensed tannins
TFA: trifluoroacetic acid
